# Prenatal Exposure to Paint Thinner Alters Postnatal Development and Behavior in Mice

**DOI:** 10.3389/fnbeh.2017.00171

**Published:** 2017-09-11

**Authors:** Hanaa Malloul, Ferdaousse M. Mahdani, Mohammed Bennis, Saadia Ba-M’hamed

**Affiliations:** Laboratory of Pharmacology, Neurobiology and Behavior (URAC-37), Faculty of Sciences Semlalia, University Cadi Ayyad Marrakech, Morocco

**Keywords:** paint thinner, prenatal exposure, fertility, reproduction, development, behavior

## Abstract

Occupational exposure and sniffing of volatile organic solvents continue to be a worldwide health problem, raising the risk for teratogenic sequelae of maternal inhalant abuse. Real life exposures usually involve simultaneous exposures to multiple solvents, and almost all the abused solvents contain a mixture of two or more different volatile compounds. However, several studies examined the teratogenicity due to industrial exposure to a single volatile solvent but investigating the teratogenic potential of complex chemical mixture such as thinner remains unexplored. This study was undertaken to evaluate developmental neurotoxicity of paint thinner using a mouse model. Mated female mice (*N* = 21) were, therefore, exposed to repeated and brief inhalation episodes of 0, 300 or 600 ppm of thinner during the entire period of pregnancy. Females weigh was recorded and their standard fertility and reproductive parameters were assessed. After birth postnatal day 1 (PND1), offspring (*N* = 88) length and body weight were measured in a daily basis. At PND5, the pups were assessed for their postnatal growth, physical maturation, reflex development, neuromotor abilities, sensory function, activity level, anxiety, depression, learning and memory functions. At adulthood, structural changes of the hippocampus were examined by estimating the total volume of the dentate gyrus. Except one case of thinner induced abortion at the higher dose, our results showed that the prenatal exposure to the solvent did not cause any maternal toxicity or decrease in the viability of the offspring. Therefore, a lower birth weight, decrease in the litter size and delayed reflexes ontogeny were registered in prenatally exposed offspring to both 300 ppm and 600 ppm of thinner. In addition, prenatally exposure to thinner resulted in increased anxiolytic- and depression-like behaviors. In contrast, impaired learning and memory functions and decreased hippocampal dentate gyrus volume were revealed only in the prenatally treated offspring by 600 ppm of thinner. Based on these results, we can conclude that prenatally exposure to paint thinner causes a long-lasting developmental neurotoxicity and alters a wide range of behavioral functions in mice. This shows the risk that mothers who abuse thinner paint expose their offspring.

## Introduction

Paint thinner is a chemical mixture of different aromatic and halogenated hydrocarbons (e.g., toluene, benzene, xylene and N-hexane), commonly used in various industrial applications as solvent in removing household paints and thinning oil-based paint (Solak et al., 2006; Verma and Gomber, 2009; Singh et al., 2012; Agin et al., 2016). Despite its usefulness, there is increasing concern about occupational exposure of industrial workers to this solvent (Yilmaz et al., 2001). In addition, thinner acts as psychoactive compounds when inhaled and its inhalation by young sniffers to achieve an euphoric state continues to be a significant worldwide health problem (Cruz, 2011; Howard et al., 2011; Bowen and Cruz, 2012).

While the volatile substance abuse is documented by diverse groups throughout the world, no epidemiological studies on inhalant abuse in Africa are available. This hampers the estimation of the extent of inhalant abuse across the general population. In the United States alone, the National Survey on Drug Use and Health has documented about 22 million 12 years old Americans and older deliberately abused inhalants at least once in their life from 2002 to 2005 (Substance Abuse and Mental Health Services Administration, 2007). Data show evidence that this abuse of inhalants has dramatically increased over the past years (Bowen and Hannigan, 2013). From 2009 to 2010, the median age at first use among those aged 12–49 years was unchanged (16.9 and 16.3 years, respectively) with 68.4% being under age of 18 years (Substance Abuse and Mental Health Services Administration, 2011). In 2013, 6.3% of teens in the United States (average age of 19.2 years) reported that the first illicit drug they had ever used was the inhalant (Substance Abuse and Mental Health Services Administration, 2014). Whereas in the past, the abuse of volatile substances was once predominantly limited to males, the gap between males and females is currently reversed: more than 50% of chronic solvents abusers are young women of reproductive age (Substance Abuse and Mental Health Services Administration, 2007; Butland et al., 2012; Bowen and Hannigan, 2013). Further, while a subcategory of inhalant abusers continue to do so into adulthood (Williams and Storck, 2007; Substance Abuse and Mental Health Services Administration, 2014), there is increasing concern about the potential negative consequences of deliberately inhaled solvents on the unborn (Bukowski, 2001; Hannigan and Bowen, 2010).

In comparison to inhalant abuse, reports of occupational exposure are not well documented due to potential limitations including small sample size, selection bias, uncontrolled and/or unspecified co-drug exposures and multiple solvent exposures (Hannigan and Bowen, 2010). Standards for a permissible exposure limit (PEL) for toluene have been established by U.S. Occupational Safety and Health Administration (OSHA) at 100 ppm (375 mg/mm^3^, calculated over an 8 h day as a time weighted average; Donald et al., 1991). After acute exposures of at least 500 ppm, a sense of euphoria generally is achieved; inhibition, confusion, incoordination, auditory and visual hallucinations occur at 600–800 ppm (Brozosky and Winkler, 1965). In contrast to chronic low-level (500 ppm) of occupational solvent exposure (Agency for Toxic Substances and Disease Registry (ATSDR) (2000)), inhalant abusers usually inhale very repeatedly, deeply and rapidly. Therefore, abused toluene minimally reaches 800 ppm, an intoxicating exposure (Filley et al., 2004), although abuse levels in chronic abusers often are higher and exceeding 5000 ppm (50 times the OSHA PEL; Ron, 1986).

In human adults, effects observed after long-term occupational exposures to thinner include dizziness, memory deficits, dementia, depression and fatigue (Kishi et al., 1993; Wang and Chen, 1993; Lee et al., 2003; Bowen et al., 2006). These wide ranges of neurobehavioral symptoms were reported to be due to the cerebral cortical and hippocampal atrophy and to the decreased brain volume observed in solvent abusers (Fornazzari et al., 1983; Lazar et al., 1983; Zur and Yule, 1990; Kamran and Bakshi, 1998). Some of these effects have been reproduced in animal studies. Neonatal exposure to volatile inhalants leads also to persistent impacts on behaviors and brain-structural properties (Benignus, 1981; von Euler et al., 1993, 2000). However, there is a limited amount of literature on teratogenic impact of prenatal exposure to paint thinner.

Placental transfer of toluene and xylene, the largest constituents of thinner mixture (Fifel et al., 2014), has been shown in both humans and rodents (Ghantous and Danielsson, 1986; Goodwin, 1988; Hass et al., 1995). Most studies demonstrated that toluene abuse during pregnancy can result in developmental disabilities and physical anomalies in the offspring (Hersh et al., 1985; Goodwin, 1988; Hersh, 1989; Arnold et al., 1994; Arai et al., 1997). Both clinically and in animal models, maternal exposure to solvents like toluene can lead to fetal or infant death (Wilkins-Haug and Gabow, 1991; Arnold et al., 1994) and a clear evidence of crude morphological teratogenicity was reported in surviving neonates (Ng et al., 1992; Hannigan and Bowen, 2010; Bowen, 2011). The neonates are small for gestational age and microcephalic with distinct dysmorphology (e.g., spatulate fingertips, short palpebral fissures, small face, micrognathia, low-set ears, deep-set eyes and hypoplastic fingernails; Hunter et al., 1979; Toutant and Lippmann, 1979; Hersh et al., 1985; Goodwin, 1988; Hersh, 1989; Arnold et al., 1994). With age, prenatal toluene exposure results in developmental delay and behavioral disruptions (Bukowski, 2001; Hannigan and Bowen, 2010; Bowen and Hannigan, 2013).

Almost, all the studies outlined above have evaluated teratogenic potential of a single volatile solvent, toluene or xylene. However, environmental and occupational exposure to solvents involves simultaneous exposure to multiple toxicants. Based hereupon, the aim of our study was to investigate the developmental and neurobehavioral consequences of prenatal exposure to thinner. The present study used a pattern of brief and repeated thinner inhalation during gestation in mice was designed to assess the developmental neurotoxicity of thinner. A battery of behavioral tests was performed to evaluate teratogenic effect of thinner on prenatal and neonatal growth, pre-weaning behavioral development, and adult behaviors; complying with the upcoming OECD Test Guideline for Developmental Neurotoxicity Studies (OECD Test Guideline Programme, 1996).

## Materials and Methods

### Animals

Experiments were performed on male and female Swiss mice (8–10 weeks old, weighing 25–30 g) raised in the central animal care facilities of the Cadi Ayyad University, Marrakech (Morocco). Animals were housed in Plexiglas cages with wood chip bedding under controlled environmental conditions (12:12 light/dark cycle, 22 ± 2°C), with standard diet and water *ad libitum*. All animal procedures were in strict accordance with the guidelines of European Council Directive (EU2010/63). All efforts were made to minimize any animal suffering and the study met the ethical standards. The study received also the approval of the Council Committee of research laboratories of the Faculty of Sciences, Cadi Ayyad University of Marrakech.

Virgin female mice (*N* = 21) at proestrus phase were mated with male in the ratio of 2:1 overnight (12 h). The following day, the onset of pregnancy was confirmed by the observation of a vaginal plug. Confirmation of positive sperm-plug corresponded to gestational day 0 (GD0). After mating, the males were removed from the cages and the pregnant females were then assigned to one of three experimental groups in random order.

### Paint Thinner Inhalation

We exposed mice to paint thinner (Sodecso, Mohammedia, Morocco) whose chemical composition, determined by gas chromatography and single wavelength monitoring spectrometry (Chemistry Analysis and Characterization Centre, University Cadi Ayyad) in our previous study (Fifel et al., 2014), includes more than 25 distinct molecules among which the most representative are Toluene (24.46%), Xylene (15.47%), Benzene (10.67%), Dichloromethylene (6.34%) and Acetone (5.55%).

Dams were exposed daily via inhalation to either 300 ppm (*N* = 8) or 600 ppm of thinner (*N* = 5) for the whole period of gestation. An “air-only” control group (*N* = 8) was manipulated for the same period and conditions as the thinner exposure groups, but without thinner (0 ppm). The exposure procedure and apparatus have been described in detail previously (see Fifel et al., 2014). Briefly, a dam was placed into a whole-body inhalation chamber (27 × 17.5 × 13.5 cm) and 200 μl or 400 μl of liquid thinner was added to a filter paper located on a glass petri dish covered by a wire mesh on the inhalation chamber floor to obtain an estimated thinner concentration of 300 ppm or 600 ppm respectively. Exposure to thinner vapor occurred twice a day between (first exposure between 8:00–9:00 a.m. and second exposure 8 h later), two sessions of 15 min (filter paper and liquid thinner were renewed at the beginning of each session), separated by 5 min interval in which mice were returned to the home cage (30 min of one exposure session and 60 min of total daily exposures).

### Maternal Observation

During the whole period of gestation, pregnant mice were observed daily in order to detect any changes in behavior, symptoms of poisoning or signs of morbidity. Abortion or premature delivery and body weight were recorded daily from GD0 until delivery. In addition, different parameters of fertility and reproduction were evaluated as described by Ait-Bali et al. (2016):

- Pregnancy index: (number of pregnant females/number of vaginal plug positive females) × 100
- Deliverance index: (number of females delivering/number of pregnant female) × 100
- Live-birth index : (number of offspring born alive/number of descendants delivered) × 100
- Viability index: (number of living offspring at day 4 of lactation/number of living offspring delivered) × 100
- Lactation index: (number of living offspring at day 21/number of living offspring born) × 100

### Birth Measures and Developmental Assessment of the Offspring

At the day of delivery designated as postnatal day 1 (PND1), all pups from each litter (Control: *N* = 22, 300 ppm-treated: *N* = 30, 600 ppm-treated: *N* = 36) were counted, sexed base on the anogenital distance, and checked for apparent morphological anomalies (e.g., facial malformations, missing digits, etc). Litter size and body weight for each pup were measured at PND1, PND7, PND14 and PND21. The appearance of physical maturation landmarks were recorded, including pinna detachment, hair appearance, incisor eruption, and eye opening. In order to not disturb and to minimize the stress on the dam and the offspring, litters were not culled, but litter with less than four pups were not included in the behavioral tests. Subsequently, pups were subjected to the following battery of pre-weaning tests (Table 1) in order to test reflex development and neuromotor ability (Fox, 1965; Adams, 1986; Moser, 2001). These reflexologic and behavioral tests, reactive to environmental and toxic conditions with greater reliability, were used to assess the maturation of the CNS (Vaglenova et al., 2008).

**Table 1:** Postnatal behavioral test battery.

Endpoints	Tests	Age at testing
Physical development	Pinna detachment	PND4
	Hair appearance	PND6
	Incisor eruption	PND8–12
	Eyes opening	PND15
Sensory-motor development	Surface righting reflex	PND5–7–9
	Cliff avoidance	PND6
	Homing	PND9
	Negative geotaxis	PND10–12
Motor coordination	Rotarod	PND24
Motor activity	Open field	PND60
Anxiety	Open field	PND60
	Elevated plus maze	PND60
Depression	Tail suspension	PND60
	Splash	PND60
Learning and memory	Step-through inhibitory avoidance	PND60
	Puzzle box	PND60

#### Surface Righting Reflex Test

Each pup was placed in a supine position and the latency to get back on all four paws was calculated. Each pup received one trial and a maximum of 60 s was given in each trail. The maximum latency of 60 s was assigned to the pups that did not right.

#### Cliff Avoidance Test

Each pup was placed on a table edge with forepaws and snout over the edge. The time spent to turn 180° away from the cliff face was measured and cliff avoidance was recorded for a maximum time of 60 s.

#### Homing Test

For 30 min in one holding cage, the pups were separated from the dam and kept at a temperature of 35°C. The litter was then transferred to a Plexiglas cage (36 × 20 cm, walls 18 cm high) containing bedding from the home cage evenly distributed on one side (14 × 20 cm, nest area) and the rest of the cage covered with clean wood shavings. Each pup was placed in the middle of the cage and video recorded for 4 min. Homing performance was scored for the time spent in the area with nest litter.

#### Negative Geotaxis Test

Pups were placed on 45° inclined plywood surface and the time taken to turn 180° from a head-downward position to face-upward was calculated.

#### Rotarod Test

Rotarod was used to evaluate the ability of mice to remain their balance on revolving rod (15 rpm) during 5 min trial. The apparatus consisted of horizontal textured roller of 3 cm diameter placed at a height of 28 cm with automatic fall detection. All mice underwent two trials with a 15 min inter-trial interval. The latency to fall off was measured for each session. The apparatus was cleaned after each trial. Litters remained with their dams until weaning on PND21 when all offspring were re-housed in groups of five same-sex littermates or other mice from the same prenatal thinner group. All animals were unhandled between PND24 and PND60 when the behavioral tests began.

### Adult Neurobehavioral Evaluation

All animals were tested at PND60 (Table 1). To assess locomotor activity and anxiety-like behaviors, we used the open field test (OFT; Wilson et al., 1976) and the elevated plus maze test (EPMT; Torres and Escarabajal, 2002; Lapiz-Bluhm et al., 2008); depressive-like state of mice was evaluated by means of the tail suspension test (TST; Steru et al., 1985) and Splash test (ST; Willner, 2005; Isingrini et al., 2010); memory retention was assessed by the step-through passive avoidance task (SPAT; Lo et al., 2009), and executive function by the Puzzle box (PB; Ben Abdallah et al., 2011). The OFT and EPMT were recorded and analyzed using Ethovision XT Noldus 8.5 video tracking program (Noldus, Netherlands) connected to a video camera (JVC). The behaviors in TST, ST, SPAT and PB were video-recorded (Samsung SCO-2080R) and measured manually using the event-recording function in the video-tracking software (Debut video capture software, NHC). All the behavioral tests were performed between 8:00 and 12:00 a.m. to avert any circadian related fluctuation in the performance of the animals. Before each mouse was introduced, the apparatus in all behavioral tests cleaned with a 75% ethanol solution to remove any trace of odor.

#### Open-Field Test

The apparatus used for this test consisted of a simple square field (50 × 50 × 50 cm). A 75W lamp was placed in porthole diffusing light and located at 200 cm from the device allowing the center of the apparatus to be under a dim light (100 lx). At the beginning of each session, mice were placed in the central part (15 cm × 15 cm) of the arena and the total distance moved, velocity, and the total time spent into center were determined over a 10 min period. The center zone is 17.5 cm from the wall of the maze, corresponding to the standard area (Park et al., 2015).

#### Elevated Plus Maze Test

The maze composed by two open arms (50 × 5 cm) and two enclosed arms (50 × 5 × 15 cm connected to a common central platform (5 × 5 cm). The maze floor and the side/end walls (15 cm height) of the enclosed arms were made of clear Plexiglas. The apparatus was raised to 50 cm from the floor and was under an approximate brightness of 200 lx. Each mouse was placed in the center facing an open arm and left to explore the maze for a single 5 min recorded session. The percentage of the time spent in the open arms was analyzed by calculating the “time spent in the open arms” divided by the “total time spent in both the open and enclosed arms”.

#### Tail Suspension Test

Mice were suspended from a plastic rod mounted 50 cm above the surface by fastening the tail with adhesive tape. Immobility, defined as the absence of any limb or body movements, was measured during 6 min.

#### Splash Test

A 10% sucrose solution was squirted on the dorsal coat of the mouse in its home cage. Because of its viscosity, the sucrose solution dirties the mouse fur and animals initiate grooming behavior. The time spent grooming body, face and paws was recorded after applying sucrose solution for a period of 5 min.

#### Step-through Passive Avoidance Learning

The step-through inhibitory avoidance apparatus consisted of bright and dark equally sized Plexiglas compartments (28.5 × 25 × 25 cm), with independent electrical grid floor, and connected by an opening guillotine door (10 × 8.5 cm). During the training session, mice were placed individually in the light chamber. Then as soon as the animal entered the dark chamber, the door was lowered and an inescapable single electric foot shock (0.5 mA) was delivered by a shocker for 5 s. Ten seconds after exposure to the foot shock, the animal was removed from the chamber to its home cage. The retention of the avoidance performance was tested 24 h later. With access to the dark chamber, each mouse was placed into the light chamber without any shock during the test session and the latency to enter the dark compartment was calculated. The mice that did not enter the dark chamber during the cut-off time (180 s) were removed from the apparatus and assigned a ceiling score of 180 s. Short latencies indicate poor retention.

#### Puzzle Box

The Puzzle box is an acrylic Plexiglas arena divided into two areas by a removable barrier: a brightly-lit (300 lx) open-field area (58 × 28 × 21 cm) and a covered goal-box area (15 × 28 × 21 cm). Mice undergo a three-day protocol, consisting of nine trials (T1–T9) with three trials per day and 1.5 h of inter-trial interval, during which they were challenged to enter into the goal zone via a narrow underpass (4 cm wide). This underpass, located under the barrier, was blocked with obstacles that are increasingly difficult to overcome as testing progress. At the beginning of each trail, mouse was placed in the open-field zone facing the goal-box, and the time taken to enter the goal zone with all four paws was recorded over a period of 3 min (training and burrowing) or 4 min (plug). Three obstruction conditions were used within this task:

- The first day (training): with no obstruction presented within the underpass, and the barrier had an open door between the open-field and goal zones during T1. On T2 and T3, doorway was closed and mouse had to enter the goal zone through the underpass.
- The second day (burrowing puzzle): T4 was a repetition of T2 and T3. However, on T5 and T6 the underpass was filled with clean bedding material and mouse had to dig their way through.
- The third day (plug puzzle): T7 was a similar to T5 and T6, but on T8 and T9, the underpass was obstructed by a cardboard plug and mouse had to draw with teeth and paws to enter the goal zone.

The performance of mice in the puzzle box permits to test the capacities of mice to remove the offered barrier in each block. This sequence allow to asses native problem-solving ability (T5 and T8), and learning/ short-term memory (T3, T6 and T9), while the repetition on the next day is used as recall and solution retention to examine the long-term memory (T4 and T7).

### Histology and Dentate Gyrus Volume

Upon completion of behavioral testing (Table 1), mice were deeply anesthetized by an intraperitoneal injection of lethal dose of sodium pentobarbital (>90 mg/kg) and perfused transcardially with 0.9% saline, followed by 4% paraformaldehyde in 0.1 M phosphate buffer (pH 7.4). Brains were removed from the skull, post-fixed overnight in the same fixative solution, cryoprotected in a 30% sucrose solution, frozen and sectioned by using a freezing microtome (Leica Microsystems, Germany). Forty-micrometer thick free-floating coronal serial sections of the dentate gyrus (DG) were collected in multiwell dishes. Sections were mounted on gelatine-coated slides and stained with Cresyl violet.

With regards to the experimental group, all area counts were conducted blind. To estimate the volume of DG, area measurement of each DG blade were made through sections of the entire DG (positioned at anterior, medial and posterior levels) at low-magnification (×6.3) by using Olympus BH-2 microscope equipped with an Olympus DP71 camera. The reconstruction of a complete photomicrograph association of different shots and their treatment has been made through image processing software Adobe Photoshop CC 2014. The area of each section was calculated by ImageJ software and the total volume of the DG was estimated by applying the Cavalieri method (Prakash et al., 1994). For each animal, 10 DG sections (along the rostrocaudal axis) were used in each analysis.

### Statistical Analysis

Statistical analyses were performed using Prism 5.0 for Windows (GraphPad software). For statistical evaluations of the different dependent variables, one-way analysis of variance (ANOVA) and two-way ANOVA followed by a Bonferroni *post hoc* for multiple comparisons were used. A difference was considered statistically significant at *P* ≤ 0.05.

## Results

### Effect of Thinner Exposure on Fertility and Reproduction Parameters of Pregnant Mice

Maternal data for the 21 dams in the three prenatal treatment groups are presented in Table 2. No clinical signs of toxicity were observed in the dams during the exposure period, except a one case of thinner induced abortion and two cases of preterm births at the higher dose of thinner (600 ppm). There were no significant differences in gestation period and gestation index between the treated groups and the control group (Table 2). Moreover, Delivery, live-birth, viability and lactation index of treated groups did not differ compared to the control group (Table 2).

**Table 2:** Pregnancy data and maternal characteristics.

Parameters	Thinner (ppm)		
	Control	300	600
**Fertility parameters**			
Number of plug vaginal positive females	10	13	9
Number of pregnant females	8	8	5
Number of aborted mice	0	0	1
Pregnancy index (%)	80	61.53	55.55
Gestation period (days)	19.05 ± 1.92	18.75 ± 1.03	18.01 ± 0.60
**Reproduction parameters**			
Deliverance index (%)	100	100	80
Live-birth index (%)	95.55	90	72.55
Viability index (%)	68.57	66.03	65.31
Lactation index (%)	68.18	60.37	51.72
Number of pups/litter	7.01 ± 2.34	6.62 ± 2.66	8 ± 1.50
**Maternal weight (g)**			
Weight gain at GD7	2.69 ± 1.30	2.79 ± 1.47	2.17 ± 1.25
Weight gain at GD15	8.09 ± 2.34	6.32 ± 1.91	5.28 ± 2.15
Weight gain at GD18	11.04 ± 3.05	9.21 ± 3.65	7.01 ± 2.40*
Weight gain between GD0–GD18	21.62 ± 2.81	16.08 ± 2.65***	14.34 ± 1.91***

Data are given as means ± SD. *P < 0.05 and ***P < 0.001 compared to control.

Concerning the body weight of pregnant females, the statistical analysis by two-way ANOVA was performed between-subject variables: treatment and gestation duration. This analysis showed a significant effect of treatment (*F*_(2,72)_ = 15.79, *P* < 0.001) and gestation period (*F*_(3,72)_ = 135.2, *P* < 0.001) on dam weights. Bonferroni *post hoc* analyses revealed that dams exposed to 600 ppm of thinner gained significantly less weight than the control at GD18 (*t* = 2.95, *P* < 0.05); otherwise, there was a significant less weight gain for dams in all treated groups compared to control group from GD0 to GD18 (control group vs. 300 ppm group: *t* = 4.61, *P* < 0.001; control group vs. 600 ppm group: *t* = 5.31, *P* < 0.001; 300 ppm group vs. 600 ppm group: *t* = 1.12, NS).

### Effect of Prenatal Exposure to Thinner on Physical Development

#### Morphological Evaluation

By using a comparative atlas of external malformations in laboratory animals and humans (Roux, 2003), each pup was observed for signs of malformations and abnormal morphological changes. No obvious external malformations were noted in the three prenatal treatment groups.

#### Body Weights of Pups

A two-way ANOVA analysis was performed considering treatment and age as main factors. Our results indicated a significant effect of treatment, age of the pups and the interaction of these two factors on body weight (*F*_(2,288)_ = 83.35, *P* < 0.001; *F*_(3,288)_ = 378.70, *P* < 0.001; *F*_(6,288)_ = 9.53, *P* < 0.001; respectively). While no significant main effects were observed for thinner prenatal exposure on PND1 body weight for all pups (*P* > 0.50, NS), Bonferroni *post hoc* analysis showed that there was a significant loss in the body weight of prenatally treated offspring compared to the control at PND7 (*t* = 0.67, NS; *t* = 5.71, *P* < 0.001; respectively), PND14 (*t* = 3.03, *P* < 0.01; *t* = 7.29, *P* < 0.001; respectively), and PND21 (*t* = 4.18, *P* < 0.001; *t* = 10.44, *P* < 0.001; respectively). Additionally, 600 ppm pretreated group also weighs less than 300 ppm pretreated group at PND7 (*t* = 5.46, *P* < 0.001), PND14 (*t* = 4.78, *P* < 0.001), and PND12 (*t* = 6.99, *P* < 0.001; Table 3).

**Table 3:** Litter and offspring characteristics.

Parameters	Thinner (ppm)		
	Control	300	600
**Offspring body weight (g)**			
Body weight at PND1	1.51 ± 0.23	1.58 ± 0.58	1.31 ± 0.25
Body weight at PND7	4.90 ± 1.02	4.71 ± 1.04	3.11 ± 1.20***^###^
Body weight at PND14	6.80 ± 1.03	5.91 ± 1.45**	4.51 ± 1.21***^###^
Body weight at PND21	8.56 ± 2.01	7.32 ± 0.07***	5.27 ± 1.15***^###^
**Offspring body length (cm)**			
Body length at PND1	4.15 ± 0.39	4.27 ± 0.33	3.89 ± 0.39
Body length at PND7	7.24 ± 0.34	6.81 ± 0.61	6.40 ± 0.75***
Body length at PND14	8.57 ± 0.28	8.54 ± 0.21	7.54 ± 1.13***^###^
Body length at PND21	10.94 ± 0.86	10.53 ± 0.69	8.69 ± 1.38***^###^
**Litter sex ratio (% males)**	52.82 ± 0.59	58.73 ± 0.39	62.48 ± 0.33
**Developmental landmarks (%)**			
Pinna detachment at PND4	100	100	90
Hair appearance at PND6	100	100	90
Eruption of lower incisors at PND8	100	100	100
Eruption of upper incisors at PND12	100	100	100
Eyes opening at PND15	100	100	88.5

Data are given as means ± SD. **P < 0.01 and ***P < 0.001 compared to control. ^###^P < 0.001 compared to treated group.

#### Body Length of Pups

There was a significant main effect of thinner exposure on body length of pups revealed by two way ANOVA analysis with treatment and age as factors (treatment: *F*_(2,288)_ = 51.52, *P* < 0.001; age: *F*_(3,288)_ = 694.30, *P* < 0.001; treatment × age: *F*_(6,288)_ = 7.64, *P* < 0.001). *Post hoc* analysis showed that no significant main effects were found for 300 ppm prenatal inhalation on body length compared to control from PND1 to PND21 (Table 3). However, prenatal exposure to 600 ppm of thinner led to a decrease in body length at PND7 (600 ppm vs. control: *t* = 3.95, *P* < 0.001; 600 ppm vs. 300 ppm: *t* = 1.98, NS), PND14 (600 ppm vs. control: *t* = 4.28, *P* < 0.001; 600 ppm vs. 300 ppm: *t* = 4.36, *P* < 0.001) and PND21 (600 ppm vs. control: *t* = 9.16, *P* < 0.001; 600 ppm vs. 300 ppm: *t* = 8.22, *P* < 0.001); no significant difference was observed at PND1 (Table 3).

#### Body Development

As reported in Table 3, no significant changes in indices of postnatal maturation were noted for pinna unfolding, hair appearance, incisor eruption, or age of eyes opening for thinner-treated animals compared to control-treated animals.

### Effect of Prenatal Exposure to Thinner on Sensorimotor Development

#### Surface Righting Reflex Test

**Figure 1:**
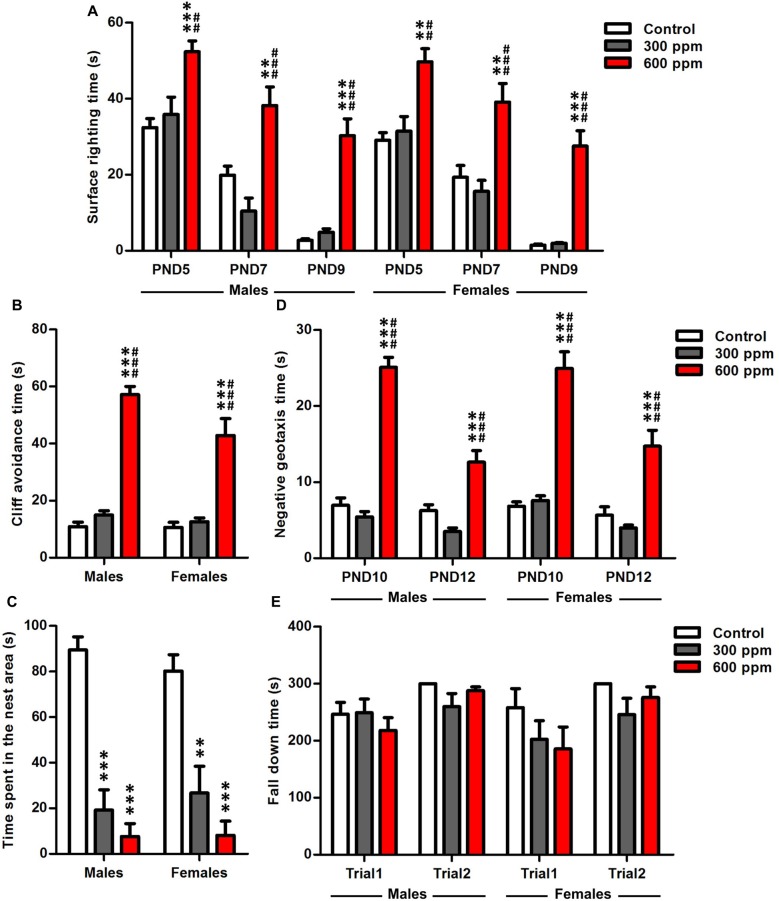
Effect of prenatal exposure to thinner on sensory-motor development. **(A)** Surface righting reflex at PND5, PND7 and PND9. **(B)** Cliff avoidance reflex at PND6. **(C)** Olfactory discrimination at PND9. **(D)** Negative geotaxis reflex at PND10 and PND12. **(E)** Motor coordination at PND24. Results are presented as mean ± SEM (Males: *N* = 13–14, Females: *N* = 8–11). **P < 0.01 and ***P < 0.001 in comparison with control group. ^##^*P* < 0.01 and ^###^*P* < 0.001 in comparison with treated group.

Data analysis by two-way ANOVA, with treatment and gender as main factors, revealed that surface righting time was significantly affected by treatment at PND5, PND7 and PND9 (*F*_(2,64)_ = 19.87, *P* < 0.001; *F*_(2,64)_ = 23.25, *P* < 0.001; *F*_(2,64)_ = 61.56, *P* < 0.001; respectively), while the sex (*F*_(1,64)_ = 1.48, *P* = 0.23; *F*_(1,64)_ = 0.34, *P* = 0.56; *F*_(1,64)_ = 1.07, *P* = 0.30; respectively) and interaction of treatment and sex (*F*_(2,64)_ = 0.03, *P* = 0.97; *F*_(2,64)_ = 0.29, *P* = 0.75; *F*_(2,64)_ = 0.06, *P* = 0.95; respectively) had no effect. To analyze more specifically the effects in both sexes of the maternal exposure to thinner, we tested the male and female data separately with two-way ANOVA by using treatment and age as main factors. The statistical analysis showed a significant main effect of treatment and age as shown by the improved surface righting times of animals between PND5 and PND9 [males (*F*_(2,114)_ = 47.13, *P* < 0.001; *F*_(2,114)_ = 53.78, *P* < 0.001; respectively); females (*F*_(2,78)_ = 45.70, *P* < 0.001; *F*_(2,78)_ = 48.33, *P* < 0.001; respectively)]. Maternal exposure to 600 ppm delayed significantly the development of offspring righting reflex compared to control and 300 ppm prenatally exposed pups at PND5 [males (*t* = 4.28, *P* < 0.001; *t* = 3.48, *P* < 0.01; respectively); females (*t* = 4.12, *P* < 0.01; *t* = 3.96, *P* < 0.01; respectively)], PND7 [males (*t* = 3.51, *P* < 0.01; *t* = 5.21, *P* < 0.001; respectively); females (*t* = 3.52, *P* < 0.01; *t* = 4.54, *P* < 0.001; respectively)] and PND9 [males (*t* = 7.28, *P* < 0.001; *t* = 6.59, *P* < 0.001; respectively); females (*t* = 7.22, *P* < 0.001; *t* = 7.68, *P* < 0.001; respectively)] (Figure 1A).

#### Cliff Avoidance Test

A significant main effect of prenatal thinner exposure was found on neonatal reflexes, as evaluated by cliff avoidance [males (*F*_(2,38)_ = 150.9, *P* < 0.001); females (*F*_(2,26)_ = 22.94, *P* < 0.001)]. Six hundred parts per million pretreated males and females mice take more time to avoid the cliff than controls and 300 ppm pretreated mice at PND6 [males (*t* = 15.76, *P* < 0.001; *t* = 14.09, *P* < 0.001; respectively); females (*t* = 5.78, *P* < 0.001; *t* = 5.89, *P* < 0.001; respectively)] (Figure 1B). Moreover, a two-way ANOVA analysis was performed considering treatment and sex as main factors. This analysis revealed that cliff avoidance time was significantly affected by treatment, sex and the interaction of these two factors (*F*_(2,64)_ = 116.2, *P* < 0.001; *F*_(1,64)_ = 5.80, *P* < 0.05; *F*_(2,64)_ = 3.54, *P* < 0.05; respectively).

#### Homing Test

The results showed that the prenatally treated mice presented a retardation in general development in the homing test at PND9 [males (*F*_(2,38)_ = 42.55, *P* < 0.001); females (*F*_(2,26)_ = 14.66, *P* < 0.001)] (Figure 1C), where a decreased time spent in the nest area was observed with respect to control in both sexes [males (300 ppm vs. control: *t* = 7.21, *P* < 0.001; 600 ppm vs. control: *t* = 8.55, *P* < 0.001); females (300 ppm vs. control: *t* = 3.99, *P* < 0.01; 600 ppm vs. control: *t* = 5.28, *P* < 0.001)]. Therefore, there is a significant effect only between groups (*F*_(2,64)_ = 51.91, *P* < 0.001) independently of sex.

#### Negative Geotaxis Test

A two way ANOVA analyses with treatment and sex as factors showed that the performance of mice was affected significantly by the experimental group independently of sex at PND10 and PND12 (*F*_(2,64)_ = 150.3, *P* < 0.001; *F*_(2,64)_ = 37.41, *P* < 0.001; respectively). To obtain more information about the effect of treatment with the age in each sex, we applied two-way ANOVA considering treatment and age as main factors. There was a significant overall main effect of prenatal thinner treatment, age and the interaction of these two factors in the negative geotaxis test [males (*F*_(2,76)_ = 110.5, *P* < 0.001; *F*_(1,76)_ = 34.88, *P* < 0.001; *F*_(2,76)_ = 19.27, *P* < 0.001; respectively); females (*F*_(2,52)_ = 64.53, *P* < 0.001; *F*_(1,52)_ = 19.05, *P* < 0.001; *F*_(2,52)_ = 5.53, *P* < 0.01; respectively)]. All prenatally 600 ppm treated pups reduced their latencies to turn 180° in comparison with control and 300 ppm pretreated animals at PND10 [males (*t* = 12.41, *P* < 0.001; *t* = 13.21, *P* < 0.001; respectively); females (*t* = 8.71, *P* < 0.001; *t* = 9.07, *P* < 0.001; respectively)] and PND12 [males (*t* = 4.36, *P* < 0.001; *t* = 6.13, *P* < 0.001; respectively); females (*t* = 4.47, *P* < 0.001; *t* = 5.75, *P* < 0.001; respectively)] (Figure 1D).

#### Rotarod Test

The two-way ANOVA analysis revealed that the motor coordination was significantly affected by the trial in both sexes [males (*F*_(1,76)_ = 8.86, *P* < 0.01); females (*F*_(1,52)_ = 5.83, *P* < 0.05)], while the treatment [males (*F*_(2,76)_ = 0.76, *P* = 0.47); females (*F*_(2,52)_ = 2.06, *P* = 0.14)] and interaction of treatment and trial [males (*F*_(2,76)_ = 1.38, *P* = 0.26); females (*F*_(2,52)_ = 0.43, *P* = 0.65)] had no effect. At PND24, our results of Rotarod test showed that no significant differences were observed between exposed and control offspring in the fall latency at trial1 [males (300 ppm vs. control: *t* = 0.08, NS; 600 ppm vs. control: *t* = 0.91, NS); females (300 ppm vs. control: *t* = 1.09, NS; 600 ppm vs. control: *t* = 1.39, NS)] and trail2 [males (300 ppm vs. control: *t* = 2.31, NS; 600 ppm vs. control: *t* = 0.65, NS); females (300 ppm vs. control: *t* = 1.71, NS; 600 ppm vs. control: *t* = 0.75, NS)] (Figure 1E).

### Effect of Prenatal Exposure to Thinner on Adults Behaviors

At PND60, the boy weight was significantly less in female (control = 24.72 ± 1.11 g; 300 ppm = 24.40 ± 1.11 g; 600 ppm = 23.72 ± 1.12 g) than male mice (control = 29.24 ± 1.03 g; 300 ppm = 27.47 ± 1.07 g; 600 ppm = 27.78 ± 1.03 g). The two way ANOVA analysis show that the difference was only significant between sexes (*F*_(1,76)_ = 19.349, *p* < 0.001), whereas the treatment and interaction sex × treatment have no effect on pups body weight (*F*_(2,76)_ = 0.76; *p* = 0, 472; *F*_(1,76)_ = 0.23; *P* = 0.79; respectively).

#### Locomotor Activity

**Figure 2:**
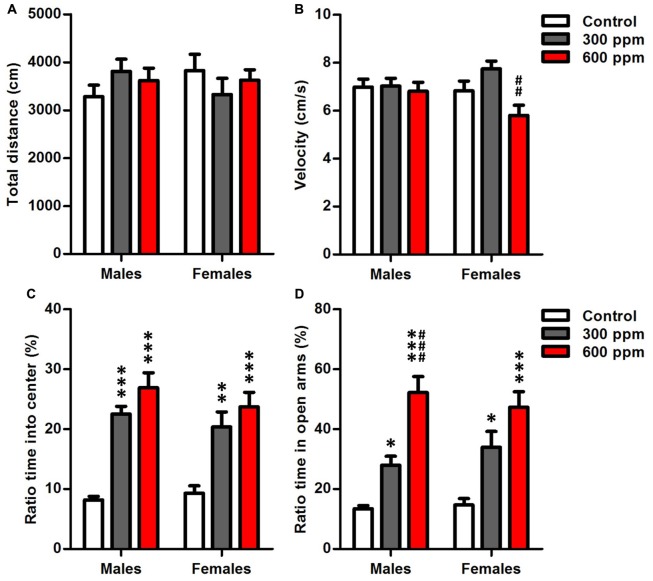
Effect of prenatal exposure to thinner on locomotor activity and anxiety-like behavior. **(A)** Total distance moved in the open field. **(B)** Moving velocity in the open field. **(C)** Ratio time spent in center of the open field. **(D)** Ratio time spent in open arms of the elevated plus maze. Results are presented as mean ± SEM (Males: *N* = 13–14, Females: *N* = 8–11). *P < 0.05, **P < 0.01 and ***P < 0.001 in comparison with control. ^##^*P* < 0.01 and ^###^*P* < 0.001 in comparison with treated group.

Open-field analysis quantified overall locomotor activity showed that mice exposed prenatally to thinner displayed no differences in distance moved from non-exposed controls in both sexes [males (*F*_(2,38)_ = 1.12, *P* = 0.33); females (*F*_(2,26)_ = 0.67, *P* = 0.52)] (Figure 2A). However, there was a significant main effect of prenatal thinner treatment on the speed of movements only in females [males (*F*_(2,38)_ = 0.11, *P* = 0.89); females (*F*_(2,26)_ = 6.79, *P* < 0.01)]; *Post hoc* analyses revealed that the 600 ppm dose group of females showed a significant decrease in velocity over the 10 min compared to 300 ppm dose group (*t* = 3.69, *P* < 0.01). Otherwise, the velocity did not differ between both prenatally exposed females and controls (Figure 2B). On the other hand, two way ANOVA analyses revealed that treatment, sex and the interaction treatment × sex had no significant effects on the speed of movements (*F*_(2,64)_ = 2.22, *P* = 0.07; *F*_(1,64)_ = 0.25, *P* = 0.61; *F*_(2,64)_ = 0.08, NS; respectively).

#### Anxiety

Anxiety-like traits were evaluated after prenatal thinner exposure using the OF and EPMTs (Figures 2C,D). Thinner prenatal exposure seems to cause a significant less anxiety-like behaviors in both sexes as revealed by long time spent in center of open field [males (*F*_(2,38)_ = 34.20, *P* < 0.001); females (*F*_(2,26)_ = 9.86, *P* < 0.001)] (Figure 2C) and open arms of elevated plus maze [males (*F*_(2,38)_ = 29.62, *P* < 0.001); females (*F*_(2,26)_ = 10.72, *P* < 0.001)] (Figure 2D). Furthermore, Bonferroni *post hoc* analysis showed that 300 ppm and 600 ppm dose groups spent significantly more time in the center of open field [males (*t* = 5.95, *P* < 0.001; *t* = 7.93, *P* < 0.001; respectively); females (*t* = 3.37, *P* < 0.01; *t* = 4.29, *P* < 0.001; respectively)] and the open arms of elevated plus maze [males (*t* = 2.79, *P* < 0.05; *t* = 7.62, *P* < 0.001; respectively); females (*t* = 2.79, *P* < 0.05; *t* = 4.63, *P* < 0.001; respectively)] compared to control group. Moreover, a significant difference was revealed between prenatally treated groups of males in EPMT (*t* = 4.68, *P* < 0.001). In addition, the two-way ANOVA revealed that the anxiety-like behavior of mice was affected significantly by the experimental group in OFT (*F*_(2,64)_ = 38.65, *P* < 0.001) and EPMT (*F*_(2,64)_ = 36.18, *P* < 0.001) independently of sex.

#### Depression

**Figure 3:**
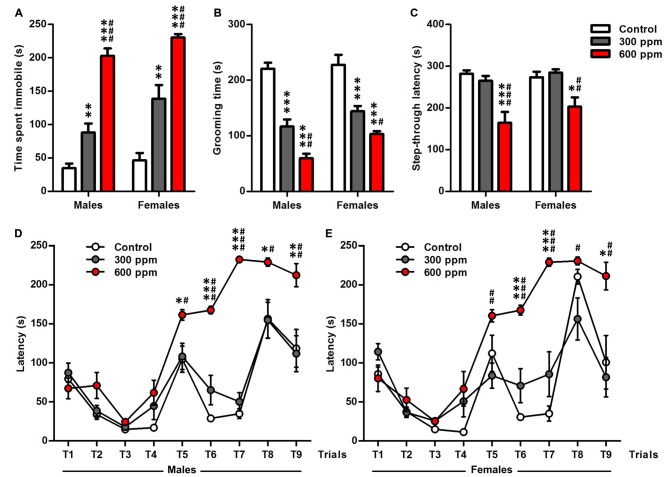
Effect of prenatal exposure to thinner on depression-like behavior, memory retention and executive function. **(A)** Time of immobility measured during tail suspension test. **(B)** Grooming time calculated during splash test. **(C)** Latency to enter the dark chamber calculated during step-through inhibitory avoidance test. **(D,E)** Latency scored to reach the goal zone of males and females during the nine trials of the Puzzle box test. Results are presented as mean ± SEM (Males: *N* = 13–14, Females: *N* = 8–11). *P < 0.05, **P < 0.01 and ***P < 0.001 in comparison with control group. ^#^*P* < 0.05, ^##^*P* < 0.01 and ^###^*P* < 0.001 in comparison with treated group.

Tail suspension test and splash test have been used to assess depression-like behavior after prenatal thinner exposure (Figures 3A,B). In tail suspension test, treated mice showed a dose dependant increase in the time of immobility compared to the control mice in both sexes [males (one-way ANOVA: *F*_(2,38)_ = 64.83, *P* < 0.001; Bonferroni *post hoc*: 300 ppm vs. control, *t* = 3.47, *P* < 0.01; 600 ppm vs. control, *t* = 11.15, *P* < 0.001; 600 ppm vs. 300 ppm, *t* = 7.48, *P* < 0.001); females (one-way ANOVA: *F*_(2,26)_ = 31.09, *P* < 0.001; Bonferroni *post hoc*: 300 ppm vs. control, *t* = 4.03, *P* < 0.01; 600 ppm vs. control, *t* = 7.87, *P* < 0.001; 600 ppm vs. 300 ppm, *t* = 4.26, *P* < 0.001)] (Figure 3A). Similarly, thinner-treated groups as compared to control group exhibited a shorter duration of grooming during splash test in both sexes [males (one-way ANOVA: *F*_(2,38)_ = 60.62, *P* < 0.001; Bonferroni *post hoc*: 300 ppm vs. control, *t* = 6.89, *P* < 0.001; 600 ppm vs. control, *t* = 10.86, *P* < 0.001; 600 ppm vs. 300 ppm, *t* = 3.77, *P* < 0.01); females (one-way ANOVA: *F*_(2,26)_ = 26.96, *P* < 0.001; Bonferroni *post hoc*: 300 ppm vs. control, *t* = 4.99, *P* < 0.001; 600 ppm vs. control, *t* = 7.27, *P* < 0.001; 600 ppm vs. 300 ppm, *t* = 2.59, *P* < 0.05)] (Figure 3B). Moreover, depression-like response was affected significantly by treatment and sex in tail suspension test (*F*_(2,64)_ = 96.34, *P* < 0.001; *F*_(1,64)_ = 8.29, *P* < 0.01; respectively) and splash test (*F*_(2,64)_ = 84.77, *P* < 0.001; *F*_(1,64)_ = 8.14, *P* < 0.01; respectively) as revealed by two-way ANOVA analyses.

#### Learning and Memory

We further examined mice by step-through avoidance learning and puzzle box tasks which have been recognized as useful experimental paradigms for assessing learning, memory retention and executive function (Figures 3C–E). In step-through inhibitory avoidance test (Figure 3C), there is significant effect of treatment (*F*_(2,64)_ = 18.47, *P* < 0.001), while the sex (*F*_(1,64)_ = 1.32, *P* = 0.25) and their interaction (*F*_(2,64)_ = 0.88, *P* = 0.42) did not affect the latency to step-through as shown by two-way ANOVA analyses. Then, analyses of the male and female data separately with one-way ANOVA revealed that the prenatal thinner exposure affect the latency to step-through during the memory retention test session in both sexes [males (*F*_(2,38)_ = 13.50, *P* < 0.001); females (*F*_(2,26)_ = 7.99, *P* < 0.01)]; therefore, in 600 ppm dose groups, there was a significant decrease in step-through latency compared to control group and 300 ppm dose groups [males (*t* = 4.82, *P* < 0.001; *t* = 4.05, *P* < 0.001, respectively); females (*t* = 3.01, *P* < 0.05; *t* = 3.76, *P* < 0.01, respectively)].

Figures 3D,E illustrate the time taken to enter the goal box in thinner-treated and control males and females mice among all trials (T1–T9) of puzzle box. Two way ANOVA showed a main effect in both sexes of treatment [males (*F*_(2,342)_ = 72.34, *P* < 0.001); females (*F*_(2,234)_ = 40.22, *P* < 0.001)], trials [males (*F*_(8,342)_ = 45.35, *P* < 0.001); females (*F*_(8,234)_ = 31.82, *P* < 0.001)] and their interaction [males (*F*_(16,342)_ = 7.48, *P* < 0.001); females (*F*_(16,234)_ = 5.66, *P* < 0.001)]. Mice of all groups similarly learned the puzzle task at the first day (T1–T3) and T4 as shown by their improved latencies on the repeated trials. Further, *post hoc* analysis displayed that all 600 ppm pre-exposed males and females mice spend more time to solve the obstacles in comparison with control and 300 ppm pre-exposed mice at T5 [males (*t* = 2.88, *P* < 0.05; *t* = 2.66, *P* < 0.05, respectively); females (*t* = 2.05, NS; *t* = 3.53, *P* < 0.01, respectively)], T6 [males (*t* = 9.34, *P* < 0.001; *t* = 6.76, *P* < 0.001, respectively); females (*t* = 6.21, *P* < 0.001; *t* = 4.76, *P* < 0.001, respectively)], T7 [males (*t* = 18.38, *P* < 0.001; *t* = 16.50, *P* < 0.001, respectively); females (*t* = 6.66, *P* < 0.001; *t* = 5.35, *P* < 0.001, respectively)], T8 [males (*t* = 2.66, *P* < 0.05; *t* = 2.68, *P* < 0.05, respectively); females (*t* = 0.37, NS; *t* = 2.94, *P* < 0.05, respectively)] and T9 [males (*t* = 3.19, *P* < 0.01; *t* = 3.36, *P* < 0.01, respectively); females (*t* = 2.93, *P* < 0.05; *t* = 3.74, *P* < 0.01, respectively)].

### Evaluation of DG Volume after Prenatal Exposure to Thinner

**Figure 4:**
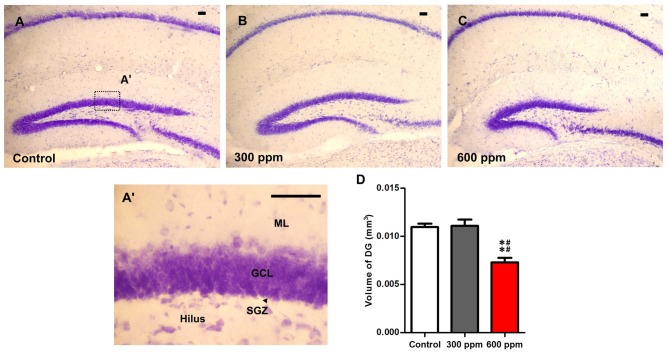
Impact of prenatal exposure to thinner on dentate gyrus volume. **(A–C)** Photomicrographs of dentate gyrus (DG) sections stained with Cresyl violet form control and prenatally treated animals by 300 ppm and 600 ppm of thinner at Bregma sections −2.18 mm. **(A’)** Photomicrograph of DG section under high magnification. **(D)** Unusual DG volume was observed in prenatally treated mice by 600 ppm of thinner. Scale bars in **(A–C,A’)** are 100 μm. Results are presented as mean ± SEM (*N* = 5). **P < 0.01 in comparison with control group and ^##^*P* < 0.01 in comparison with treated group. ML, Molecular layer; GCL, granule cell layer; SGZ, subgranular zone.

Cresyl violet-stained sections at the same Bregma levels, showed an important reduction in the entire volume of the hippocampus following prenatal exposure to paint thinner. The observed changes were more prominent with 600 ppm than those observed in 300 ppm and control (Figures 4A–C). Indeed, the analysis of the dentate gyrus volume of the hippocampus subregions by using the Cavalieri method showed a significant main effect (*F*_(2,6)_ = 18.33, *P* < 0.01). Moreover, statistical analysis among the different experimental groups revealed that the volume of DG is sharply reduced only in the 600 ppm pre-exposed mice in comparison with control and 300 ppm pre-exposed mice (*t* = 5.14, *P* < 0.01; *t* = 5.33, *P* < 0.01; respectively). Conversely, mice that were prenatally exposed to 300 ppm of thinner displayed no differences in the volume of DG as compared to control mice (*t* = 0.18, NS; Figure 4D).

## Discussion

The rationale for this study is the raise in inhalant-abuse among pregnant women, which usually include solvents containing a mixture of volatile compounds, and environmental and occupational exposures consist mostly of simultaneous exposures to various solvents. Most published works described the teratogenicity due to industrial exposure to a single volatile solvent and the teratogenic potential of chemical mixture such as thinner remains under-investigated. Hence, the current study is the first attempt evaluated developmental and behavioral effects in offspring of mice prenatally exposed briefly and repeatedly to thinner. The present results showed that, despite one case of thinner induced abortion at the higher dose, prenatal exposure to the solvent did not cause any maternal toxicity nor decrease viability of the offspring. In contrast, there were significant effects on birth weight, litter size, sensory-motor development, recognition memory, anxiolytic- and depression-like behaviors independently of sex at adult age.

### Methodological Considerations

The present study used a pattern of brief (15 min) and repeated (two sessions of 15 min twice a day, 60 min/day in total) inhalation of different concentrations of thinner (300 or 600 ppm) during pregnancy (7 days/week for 20 days) in mice. This pattern of exposure, typically found with inhaled solvent abuse, mirrors exposure levels, administration route and exposure durations. The fetuses in this study were exposed to concentrations of thinner mimicking those attained clinically. In previous study in our lab, we have used exposure parameters identical to the present study (Fifel et al., 2014). It was reported that depending on the solvents, the concentrations used during abuse episodes in human vary considerably and involves mostly high concentrations (up to 15,000 ppm; Hathaway and Proctor, 2004; Bowen et al., 2006). However, it has been demonstrated that mean solvent concentrations in the atmosphere measured in working environments in several industries (e.g., car painting, printing, fiber glass reinforced unsaturated polyester industries, and furniture industries) are in the range of 35–210 ppm (short term measurements; Dreiem et al., 2005). This indicates that the nominal concentrations used in this study are in a range that is relevant for occupational exposure; we suggested that concentrations slightly higher than 200 ppm but largely lower than the concentrations used to mimic binge patterns will lead to teratological and developmental consequences. The most common route of exposure in the working environment is by inhalation, and since the inhalant abuse is periodic or episodic rather than continuous, repeated and brief episodes of thinner exposure (4 × 15 min/day) were used. Three reasons justify our choice of this paradigm: Firstly, the analyses by gas chromatography showed that all thinner components are evaporated during the 15 min after thinner injection. Secondly, we used a static system of solvents delivery which causes the carbon dioxide (CO_2_) accumulation over time (Bowen et al., 2006). Therefore, in order to prevent potential CO_2_ poisoning, the 30 min duration of each inhalation session was divided in 2 × 15 min separated by 5 min during which the animals were returned to their home cage where they could breathe fresh air. The third reason behind using 2 × 15 min sessions is based on epidemiological data showing that, the exposure to solvents during abuse episodes in human typically lasts a few minutes (<10 min; Hathaway and Proctor, 2004; Bowen et al., 2006). In addition, the exposures to inhalants began on GD8 in several previous studies (Bowen et al., 2009; Bowen and Hannigan, 2013; Callan et al., 2015) because GD8 is post implantation and limits the risk of resorption. Since the timing of the exposure period can influence outcome, in the present animal model, the thinner exposures were performed from GD0 through GD20. This animal model, that patterns exposure to model inhalant abuse practices, will be able to assess the teratogenic potential of abused solvents with highly fidelity and sensitivity to clinical situation than other patterns.

### Effects on Reproduction Parameters

The effects of thinner exposure on offspring development and behavior occurred in the absence of any obvious maternal or fetal toxicity. Despite some cases of thinner induced abortion and preterm births at the higher dose of thinner, no significant differences were observed in fertility and reproduction parameters. Previous studies of toluene exposure in rats have produced conflicting results regarding fertility and reproduction indices (Bowen et al., 2005, 2009; Roberts et al., 2007; Bowen and Hannigan, 2013). A possible explanation for the discrepancies in the results of maternal and fetal toxicity may lie in the unique methodologies used by each experimenter with regard to routes of administration, exposure duration and concentration, stage of development during exposure, maternal age, and the species used. Clinically, several cases of inhalants-related embryopathy and malformations have been reported after solvents abuse by pregnant women (e.g., toluene, 1,1,1-trichlorethane and gasoline; Arai et al., 1997; Hannigan and Bowen, 2010; Bowen, 2011). Individual cases of perinatal death and surviving neonates, showing evidence of morphological and functional teratogenicity (termed “Fetal Solvent Syndrome”), have been reported in children born to women exposed to very high levels of toluene during their pregnancies (Arnold et al., 1994; Pearson et al., 1994).

In the present work, the daily exposure of pregnant mice to thinner vapor during gestation reduced weight gain in dams. Moreover, although body weights and length of the thinner-prenatally treated pups did not differ from non-treated pups at birth (PND1), but showed a significant decrease in the body growth from PND1 until PND21. Several studies have suggested that the current growth restrictions could be attributed to the decreased gestational body weight and possible under-nutrition of the mother (Saillenfait et al., 2007; Bowen and Hannigan, 2013; Callan et al., 2015). Others have reported that fetal and placental weights regardless of maternal food consumption decreases after prenatal exposure to solvent (Gospe et al., 1994; Gospe and Zhou, 1998). Therefore, it is possible that inhalant exposure during pregnancy may have persistent impacts on lactation and/or maternal behavior which were insufficient for postnatal maturation of the offspring.

### Postnatal Development of Pre-Exposed Pups

Following prenatal exposure to thinner, the postnatal observations showed an overall delay in the innate reflexes While there was no significant thinner-induced shift in physical maturation as evaluated by physical landmarks (i.e., pinna detachment and incisors eruption), mice offspring exposed *in utero* to high concentration of thinner showed significant reduction in postnatal growth up to weaning. These deficits were seen in measures of sensory-motor development and reflex ability. Pups exposed to the higher concentration of thinner exhibited delays in the righting reflex, cliff avoidance reflex and negative geotaxis reflex relative to sham-exposed pups. These results agreed with previous findings, which reported that the prenatal exposure to xylene and toluene delayed the development of those behavioral patterns (Hass et al., 1995; Hougaard et al., 1999; Bowen and Hannigan, 2013). This alteration observed in reflexologic tests in the exposed pups may indicates important neural damage for righting such as vestibular function (Secher et al., 2006), and also deficits accompanying a mouse cerebellum developmental delay (Aruga et al., 1998). The vestibular system functions already at birth (Altman and Sudarshan, 1975), but righting is not accomplished until after maturation of various placing and supporting reactions in the motor system. It is also noted that thinner-exposed pups displayed an altered response in homing test, which considered a test incorporates more complex measures of interest in social odors, cognitive and sensorimotor abilities (Scattoni et al., 2008).

### Neurobehavioral Effects on Adult Prenatally Exposed to Thinner

In addition to any changes in the body weight in both sexes, our data relative to performance in Rotarod test, distance moved, and moving velocity in the open-field test at PND60 showed that the animals exposed to thinner *in utero* did not differ from controls, indicating that both motor coordination and activity were not disturbed.

Interestingly, the prenatal exposure to the thinner at the used concentrations in this study induces anxiolytic- and depression-like behaviors at adulthood as assessed by different behavioral tests. These findings joined those of previous studies obtained by Bowen et al. (1996), Páez-Martínez et al. (2003) and Fifel et al. (2014) showing the anxiolytic properties of different solvents (thinner, toluene, benzene, 1,1,1-trichloroethane, diethyl ether and flurothyl) in exposed adult mice. At the molecular level, the anxiolytic effect of thinner is not an unexpected finding and it could be related to the ability of its components to act as positive modulators of GABAA receptors, like other CNS depressant molecules (Bale et al., 2005; Williams et al., 2005).

The results derived from the effects of prenatal thinner exposure on the offspring cognitive functions highlight impairments in executive function and memory retention at adult age, as revealed by the Puzzle box and the step-through inhibitory avoidance tests. The Puzzle box test, considered a highly reliable test of higher-order cognitive functioning (Ben Abdallah et al., 2011), resulted in thinner 600 ppm-exposed mice exhibiting prolonged latencies to reach the goal zone relative to control mice. Moreover, the exposure *in utero* to 600 ppm of thinner resulted in decreased latency to enter the dark chamber calculated during step-through inhibitory avoidance test. No differences were seen between thinner 300 ppm-exposed mice and their control counterparts in the latencies of both behavioral tests. These observations are compatible with findings by Hass et al. (1995, 1999) who showed that the mnemonic processes are particularly vulnerable to xylene and toluene effects. Additionally, similar results have been demonstrated by other experimental tests (i.e., the object recognition test and the Morris water maze task), which involve short-term memory or spatial learning (Fifel et al., 2014; López-Rubalcava et al., 2014; Callan et al., 2015). No mechanistic studies have been designed to find out the molecular targets implicated in cognitive impairments, but changes in the expression of NMDA receptor (subunits NR1 and NR2 mRNA), NMDA receptor antagonism, and inhibition of neurogenesis in the hippocampus could be associated with the impaired memory observed in mice exposed to thinner during gestation (Seo et al., 2010; Huerta-Rivas et al., 2012; Win-Shwe and Fujimaki, 2012).

As the DG subregion of the hippocampus is a substrate for both cognition and mood regulation (Sahay et al., 2011), it was important to evaluate whether the prenatal exposure to thinner led to any morphological alterations in this area. Histological evidence and volumetric finding indicate that thinner may interfere with the development of the hippocampus, demonstrating by the smaller volume of the granule cell layer of the hippocampal dentate gyrus in animals exposed prenatally to 600 ppm of thinner. The finding of reduced DG volume after prenatal exposure to thinner is in accord with a study on the effects of postnatal exposure to 100 and 500 ppm toluene on the developing hippocampus in rats (12 h/day, day 1–28 postnatally; Slomianka et al., 1990). The results of those authors, demonstrated that in addition of the smaller volume observed within the area dentate of exposed animals (granule cell layer, hilus, and the commissural-associational zone of the dentate molecular layer), argyrophilic cells and pronounced granule cell degeneration were found in the granule cell layer of animals exposed to the higher dose of toluene. The effects of early developmental thinner exposure on DG volume may be secondary to a number of indirect effects, including disrupted synaptogenesis (Lin et al., 2009), altered expression of neurotrophic factors (e.g., nerve growth factor and brain-derived neurotrophic factor), increases in NMDA receptors (Lee et al., 2005), pro-inflammatory cytokines and glial markers (e.g., glial fibrillary acidic protein; Win-Shwe et al., 2012). While the behavioral alterations seen in prenatally exposed mice are consistent with hippocampal involvement, causal relationship, however, remains to be elucidated.

In summary, our experiments have provided several main findings to support our hypothesis that the exposure *in utero* to paint thinner results in behavioral, functional, and structural deficits. These alterations may be irreversible as they were apparent at adult age. Real life always involves simultaneous inhalations of multiple solvents, indicating the need for further studies, with combinations of substances, on the outcomes and mechanisms of thinner-induced developmental and behavioral effects.

## Author Contributions

HM, MB, FMM and SB designed the experiments; HM and FMM performed the experiments; HM, MB, FMM and SB performed the analysis of the data; HM and FMM assembled the figures. HM, SB, MB and FMM wrote and edited the manuscript. All authors validated it.

## Conflict of Interest Statement

The authors declare that the research was conducted in the absence of any commercial or financial relationships that could be construed as a potential conflict of interest.
